# 3D bioprinting patient-derived induced pluripotent stem cell models of Alzheimer’s disease using a smart bioink

**DOI:** 10.1186/s42234-023-00112-7

**Published:** 2023-05-24

**Authors:** Claire Benwood, Jonathan Walters-Shumka, Kali Scheck, Stephanie M. Willerth

**Affiliations:** 1 Department of Mechanical Engineering, University of Victoria, Victoria, BC V8P 5C2 Canada; 2 Division of Medical Sciences, University of Victoria, Victoria, BC V8P 5C2 Canada; 3 School of Biomedical Engineering, The University of British Columbia, Vancouver, BC V6T 1Z3 Canada

**Keywords:** 3D bioprinting, Microspheres, Neural tissue, Bioink, Stem cells, Alzheimer’s disease

## Abstract

**Background:**

Alzheimer’s disease (AD), a progressive neurodegenerative disorder, is becoming increasingly prevalent as our population ages. It is characterized by the buildup of amyloid beta plaques and neurofibrillary tangles containing hyperphosphorylated-tau. The current treatments for AD do not prevent the long-term progression of the disease and pre-clinical models often do not accurately represent its complexity. Bioprinting combines cells and biomaterials to create 3D structures that replicate the native tissue environment and can be used as a tool in disease modeling or drug screening.

**Methods:**

This work differentiated both healthy and diseased patient–derived human induced pluripotent stems cells (hiPSCs) into neural progenitor cells (NPCs) that were bioprinted using the Aspect RX1 microfluidic printer into dome-shaped constructs. The combination of cells, bioink, and puromorphamine (puro)-releasing microspheres were used to mimic the in vivo environment and direct the differentiation of the NPCs into basal forebrain-resembling cholinergic neurons (BFCN). These tissue models were then characterized for cell viability, immunocytochemistry, and electrophysiology to evaluate their functionality and physiology for use as disease-specific neural models.

**Results:**

Tissue models were successfully bioprinted and the cells were viable for analysis after 30- and 45-day cultures. The neuronal and cholinergic markers β-tubulin III (Tuj1), forkhead box G1 (FOXG1), and choline acetyltransferase (ChAT) were identified as well as the AD markers amyloid beta and tau. Further, immature electrical activity was observed when the cells were excited with potassium chloride and acetylcholine.

**Conclusions:**

This work shows the successful development of bioprinted tissue models incorporating patient derived hiPSCs. Such models can potentially be used as a tool to screen promising drug candidates for treating AD. Further, this model could be used to increase the understanding of AD progression. The use of patient derived cells also shows the potential of this model for use in personalized medicine applications.

**Supplementary Information:**

The online version contains supplementary material available at 10.1186/s42234-023-00112-7.

## Background

Alzheimer’s disease (AD) is a progressive, neurodegenerative brain disorder that leads to a deterioration of cognitive function in the patients affected including a decreased capacity for normal memory, language, and behaviour. It is becoming increasingly prevalent in aging populations with the World Health Organization (WHO) estimating that worldwide approximately 12% of people over the age of 65 are affected (Chen et al. 2022). AD is characterized by the buildup of amyloid beta plaques and neurofibrillary tangles (NFT) resulting in the degeneration of the brain. Hyperphosphorylation of tau creates the NFTs along with the build-up of amyloid beta leads to the destabilization of the cytoskeleton, axonal degeneration, inflammation, and neuronal cell death (Ooi et al. 2013). Basal forebrain cholinergic neurons (BFCN), responsible for memory and spatial learning, are the first to be affected and degenerate in AD. Tau accumulation has been found in the basal forebrain cholinergic system early in the progression of AD and has been attributed to the early signs of cognitive decline in patients because of the disruption of cortical cholinergic input (Bissonnette et al. 2011).

Currently, no cure for AD exists - the FDA approved therapies only provide some symptomatic relief that provide improvements in the quality of life for patients but do not alter the progression of the disease. AD treatments include cholinesterase inhibitors that operate by blocking the enzymes that break down acetylcholine as well as prolonging its activity at cholinergic synapses (Anand and Singh 2013; Hampel et al. 2018). Another type of treatment - N-methyl-D-aspartic acid (NMDA) receptor agonists - target overly active glutamate receptors and prevent neural degeneration by decreasing the buildup of phosphorylated tau (Zhang et al. 2016). Many different clinical trials have been conducted unsuccessfully for finding a treatment that will slow or reverse the cognitive decline of patients. These approved treatments include various anti-amyloid drugs that target the different pathways of amyloid beta 42 production and aggregation including the controversial drug Aduhelm and the recently approved lecanemab (Karlawish and Grill 2021; Swanson et al. 2021). There are drugs that inhibit the kinases and activators of phosphatases to prevent the buildup of hyperphosphorylated tau (Ooi et al. 2013). Current pre-clinical models of AD include 2D cell culture models, animal models, and human cadaveric tissues. The failures and limitations of these drugs in clinical trials, however; demonstrate the need for more physiologically relevant AD models to minimize the cost and to increase the speed of drug discovery as well as provide more understanding of its pathogenic progression.

Human induced pluripotent stem cells (hiPSC) are reprogramed somatic cells that have the ability to self-renew and can be differentiated into any cell type in the human body with the use of transcription factors (Takahashi et al. 2007). hiPSCs generated from patients can model the progression of AD with the potential to identify and validate promising drug candidates (Israel et al. 2012; Lee et al. 2020; Yagi et al. 2011). Patient-derived hiPSC diseased neurons from individuals with familial Alzheimer’s disease (FAD) carrying a PS1 and a PS2 mutation have shown both an increase in amyloid beta 42 secretion as well as a response to different inhibitors compared to non-AD controls (Yagi et al. 2011). Israel et al. reported that when they generated two purified neuronal cultures from primary fibroblasts taken from patients with sporadic Alzheimer’s disease (sAD), only one of the genomes displayed significant AD phenotypes (Israel et al. 2012). This demonstrates the possibility for personalized treatments of AD as well the benefit of determining patient-specific drug responses.

Multiple studies have been conducted with different 3D models of organoids and post-mortem tissues to utilize hiPSCs to model AD (Schwartz et al. 2015; Di Lullo and Kriegstein 2017). Flamier et al. used post-mortem human samples and hiPSC-derived cortical neurons to investigate the role of BMI1 in AD and found that its addition could help prevent the buildup of tau deposits (Flamier et al. 2018). Brain organoids produced from patient-derived hiPSCs were developed by Raja et al. that showed amyloid beta aggregation and hyperphosphorylated tau proteins. When β- and γ-secretase inhibitors were applied, a reduction in amyloid and tau pathology was observed (Raja et al. 2016). Finally, five cerebral organoids with cortical neurons again derived from hiPSCs were used to show the differences between various iPSC lines generated from multiple patients (Arber et al. 2020). Differing ratios between secreted amyloid beta peptide fragments corresponded to different mutations of the cell lines (Arber et al. 2020). Zhang et al. utilized a co-axial bioprinter to create AD core shell models incorporating human neural progenitor cells (NPCs) with 2% Matrigel as the core and 2% alginate as the shell (Zhang et al. 2022). The NPCs were transduced with a lentiviral gene vector that encoded human amyloid beta precursor protein with V6421 (London) and K595 N/M596L (Swedish) mutations in order to overexpress amyloid beta (Zhang et al. 2022). It was shown that compared to 2D models, the 3D core shell models had higher levels of differentiation when stained for astrocyte and neuronal biomarkers as well as greater levels of amyloid beta aggregation and expression of tau. It was also noted that amyloid beta was identified on day 14 of the culture but not on day 2 indicating the progression of AD in the model. Finally, evidence of a physiologically relevant model was demonstrated by the self-clustering and cell interactions of the NPCs (Zhang et al. 2022). These studies highlight the importance of hiPSCs in understanding cellular mechanisms as well as their potential role in the use of personalized medicine. 3D hiPSC models of disease are a potential alternative for modelling the complexity of the human brain and to increase the success rate of new AD drugs in clinical trials because they more closely resemble what is occurring in vivo (Centeno et al. 2018).

**Fig. 1:**
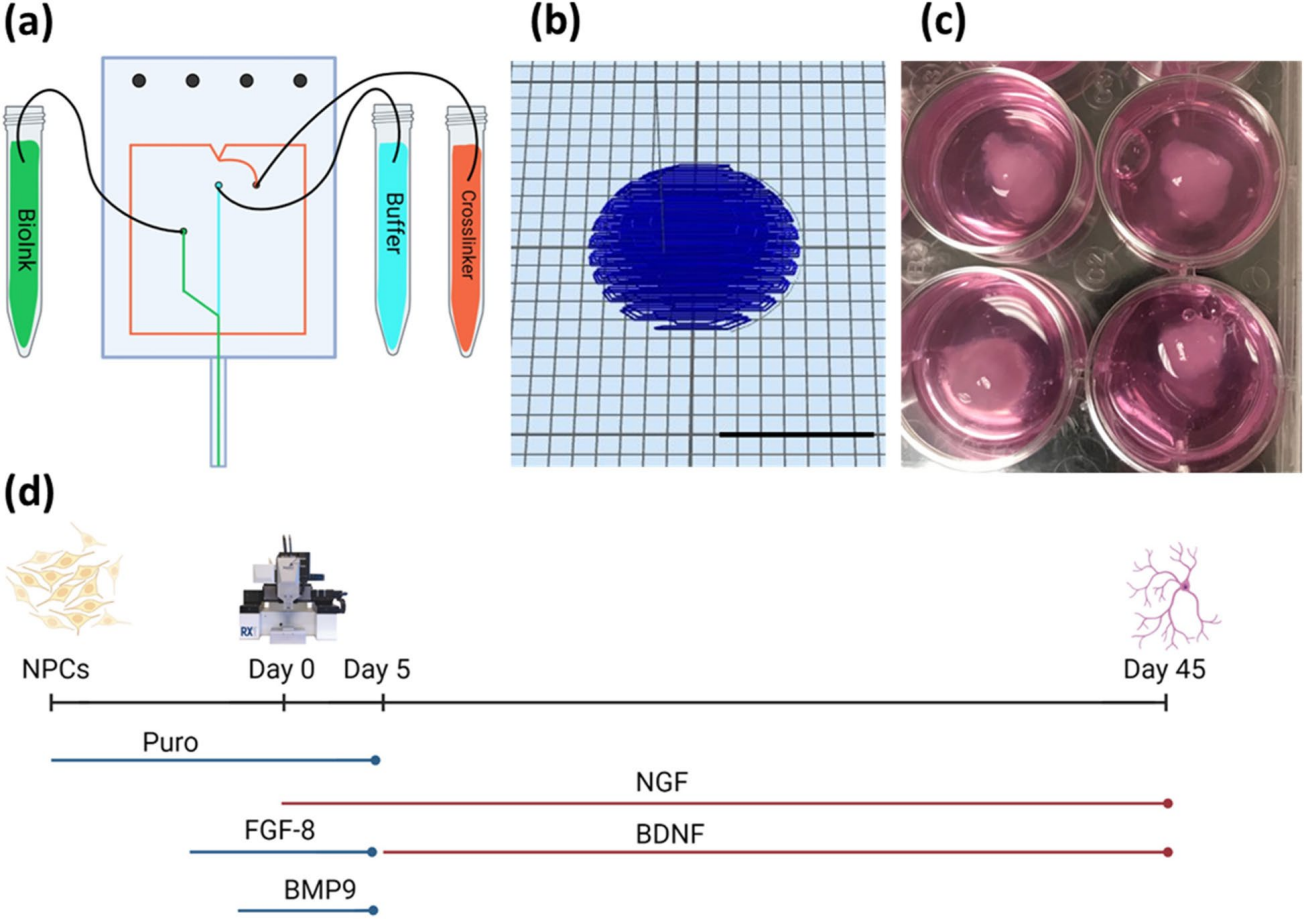
**a** Diagram of Aspect Biosystems DUO™ microfluidic printhead (created with Biorender.com). The bioink and crosslinker are extruded in separate channels before polymerization in the nozzle. **b** CAD file of the dome shaped construct bioprinted for all groups. Scale bar is 10 mm. **c** Image of four printed constructs in a 12 well plate directly after bioprinting. **d** Schematic diagram displaying the schedule of small molecules added to the culture to guide the differentiation of NPCs to BFCNs (Created with Biorender.com)

Bioprinting, an additive manufacturing technique, combines cells and biomaterials to create 3D structures that mimic in vivo tissues (Li et al. 2016). The artificial ECM environment that it creates allows for cells to survive and grow because it provides the required support and structure for the cells. The Aspect RX1 bioprinter uses a microfluidic extrusion system that allows cells to be protected from shear stress by printing at a low pressure. Their system uses Lab-on-a-printer (LOP^TM^) microfluidic printheads that are made up of multiple microscale channels able to extrude multiple materials and a crosslinker at the same time (Fig. 1). This allows for chemical crosslinking to occur, where the bioink polymerizes in the nozzle, and creates a printable hydrogel that can be extruded at a low pressure increasing the viability of the printed cells. Using this method, tissue constructs can be produced in a fast and reproducible way allowing for high throughput creation of constructs. Bioprinting has been shown to be effective at generating neural tissue models utilizing a fibrin-based bioink developed by Abelseth et al., that can promote neural differentiation and maturation (Sharma et al. 2020; Smits et al. 2020; De la Vega et al. 2021; Abelseth et al. 2018).

Microspheres are small micron-sized particles that can be incorporated into the bioink. Differentiation factors can be incorporated into their fabrication so during their degradation they will be released and help ensure the maturation of NPCs into the desired mature neuron phenotype (De la Vega et al. 2021; Agbay et al. 2018). These are valuable to be used in bioprinting because it allows for the even and slow release of growth factors over time as soluble media may not reach the centre of larger constructs (De la Vega et al. 2018). Microspheres have been incorporated into the fibrin-based bioink and patterned specifically during the bioprinting process to increase cell differentiation and enable localized drug delivery (Sharma et al. 2020; De la Vega et al. 2021). The addition of microspheres to the bioink has also led to an improvement in the mechanical properties of the bioprinted tissue constructs. The resulting increase in stiffness aids with the long- term culture and maturation of the bioprinted neural tissue as it decreases the rate of degradation of the constructs (Sharma et al. 2021a). Sharma et al. observed no negative effects of microsphere incorporation in relation to the porosity of the structure and saw increased chemical bonding between the microspheres and the polymeric chains of the biomaterials in the bioink (Sharma et al. 2021a).

The aim of this study was to use a fibrin-based bioink with Aspect Biosystems microfluidic-based extrusion printhead system to bioprint AD tissue constructs. The bioink including cells and PCL microspheres encapsulated with puro were used to print tissue constructs. Patient-derived healthy and AD NPCs were printed and then differentiated into BFCN models and evaluated on days 1, 30, and 45 for cell viability, expression of BFCN markers and AD markers, and electrical properties of the constructs. This work shows for the first time the characterization of bioprinted patient derived BFCN models after 45 days of culture. In the future, it could be used to screen promising drug candidates for the treatment of AD as well as to facilitating personalized medicine by using hiPSCs from patients with the disease.

## Methods

### Preparation of microspheres

Microspheres were fabricated as previously described using an oil-water (o/w) single-emulsion method (De la Vega et al. 2021; Agbay et al. 2018; Sharma et al. 2021b). For the oil phase PCL (Mn ~ 45,000, Sigma-Aldrich, cat. No. 704105) was added to dichloromethane (DCM) (reagent/ACS grade, VWR, cat. No. BDH23372) to obtain a concentration of 106.67 g/mL (PCL/DCM) and stirred for 15 min at 950 rpm until the solution was clear. 3 mL of a 384.5 µM puro (Cayman Chemical, cat. No. 483367-10-8) stock solution dissolved in 100% ethanol was added to achieve a puro concentration of 0.93 µg/mg (w/w puro/PCL). The water phase was prepared by adding 15 mL of 2% (w/v) polyvinyl alcohol (PVA, Mw ~ 13,000-23,000, 87%-89% hydrolyzed, Sigma-Aldrich, cat. No. 363170) to 85 mL dH_2_O and heating to between 35–39 °C while stirring at 200 rpm. After removing the oil phase from the stir plate, 3 mL of 2% PVA was added slowly to make sure that the boundary layer was not disrupted, and that the emulsification does not break before the components are fully mixed. It was then vortexed for 30 s at 3000 rpm before pouring into the side of the vortex of the 0.5% PVA and stirred at 500 rpm and 35 °C for 4 h. The microspheres were then filtered through 37 µM reversible strainers (STEMCELL Technologies cat. No. 27215) and washed seven times with dH_2_O. They were then lyophilized for 24 h and stored at -80 °C until ready for use. Before bioprinting, the microspheres were sterilized using plasma sterilization.

### Microsphere characterization

#### Size and surface analysis

Scanning electron microscopy (SEM) was used to determine the size and shape of the microspheres. The microspheres were prepared by mixing 0.1 mg of lyophilized microspheres and 50 µL of 100% ethanol. They were then mounted onto a 2 µL SEM stub and left overnight for the ethanol to evaporate. Using the Anatech Hummer VI, the microspheres were thoroughly sputter-coated with gold-palladium. Images were taken with a Hitachi S-4800 FE SEM at an accelerated voltage of 1.0 kV and a working distance of 9.4 mm. The microsphere diameters were measured using QUARTZ PCI software.

#### Encapsulation efficiency

To determine encapsulation efficiency, puro was extracted from the microspheres and quantified by high-performance liquid chromatography (HPLC) based on the protocol from Agbay et al. (2018) and De la Vega et al. (2018). To extract the puro from the microspheres, 250 µL of Acetonitrile 190, HPLC grade (Caledon Laboratory Chemicals, cat. No. 1401-7-40) was added to 10 mg of lyophilized microspheres in a 1.5 mL microcentrifuge tube. The samples were then vortexed for 30 s at 3000 rpm and then mixed for 5 min at 2500 rpm using the MixMate (Eppendorf, cat. No. 2231000804). Another 250 µL of acetonitrile was added and the mixing steps were repeated before adding 500 µL dH_2_O and mixing again. The samples were centrifuged for 5 min at 15,000 rpm before being placed in the -80 °C freezer for 5 min. Finally, the sample was centrifuged again for 5 min at 15,000 rpm. A 0.2 µm polytetrafluoroethylene (PTFE) syringe filter on a 1 mL syringe was prewet with 50 µL acetonitrile (ACN)before the supernatant from the sample was filtered into 2 mL amber HPLC vials.

An Agilent 1100 with a quaternary pump and diode array detector (DAD) was used to perform HPLC. The samples were analyzed at 300 nm using a system in direct infusion mode with no separation. The solvents used were HPLC grade acetonitrile and MilliQ H_2_O both containing 0.1% (V/V) trifluoroacetic acid (TFA) (Fisher Scientific). The runs were isocratically done at a ratio of 70%:30% respectively with an injection volume of 20 µL and flow rate of 1 mL/minute at 21 °C. ChemStation software was used to analyze the data and a calibration curve was made using a standard stock solution of puro diluted into acetonitrile. Encapsulation efficiency was determined by comparing the amount of encapsulated drug (D_encapsulated_) to the amount of drug originally added (D_theoretical_).

#### Polydispersity index

Dynamic light scattering (DLS) was performed on the microspheres using a Malvern Zetasizer Pro to determine the polydispersity index (PI). To prepare the samples, 2 mL of 100% ethanol was sterile filtered using a 0.2 µm filter and combined with 2 mg of lyophilized microspheres. A cuvette (Malvern Panalytical, cat# PCS8501) was rinsed once with filtered dH_2_O and twice with filtered 100% ethanol. 1 mL of the microsphere solution was then added to the cuvette. The samples were run in triplicate at 25 °C, an equilibration time of 120 s, and a back scatter angle of detection before analysis with ZS Explorer software. 

### hiPSC expansion and neural induction

Patient-derived AD hiPSCs sourced from a male patient with an APP gene mutation causing fAD (ADAPP) and hiPSCs from an age-matched healthy female (HN1) were cultured in mTeSR^TM^1 (STEMCELL Technologies cat no. 85850) on cell culture plates coated with Corning®Matrigel®. The hiPSCs were then passaged onto 6-well plates coated with 50 µg/mL of poly-L-ornithine (PLO, cat no. P4957, Sigma, St. Louis, MO, USA) and 10 µg/mL of laminin (cat no. L2020, Sigma, St. Louis, MO, USA). Neural induction was performed using a monolayer culture protocol and a STEMdiff™ SMADi Neural Induction Kit (STEMCELL Technologies cat no. 08581). Briefly, after the hiPSCs were plated as single cells onto 6-well plates, they were cultured for seven days at 37 °C and 5% CO_2_ with daily media changes performed using STEMdiff™ Neural Induction Medium + SMADI. On day seven the cells were again passaged onto 6-well plates and daily media changes were performed with STEMdiff™ Neural Induction Medium + SMADI. This continued until passage three on day twenty-one when the cells were ready for expansion.

### Neural progenitor cell expansion and priming

AD and HN1 NPCs were expanded with STEMdiff™ Neural Progenitor Medium (NPM) (cat no. 0583 STEMCELL Technologies) on 6-well plates coated with PLO and laminin. Full volume media changes were performed every second day. Cells were switched to STEMdiff™ Neural Induction Medium (NIM) (STEMCELL Technologies cat no. 05385) and were supplemented with 100 ng/mL purmorphamine (puro) (STEMCELL Technologies cat no. 72204) on days 1–6, 100 ng/mL puro and 100 ng/mL fibroblast growth factor 8 (FGF-8) (STEMCELL Technologies cat no. 78128) on days 6–12, and 100 ng/mL puro, 100 ng/mL FGF-8, and 10 ng/mL bone morphogenic protein 9 (BMP9) (Peprotech cat no. 120-07) on day 12 with half volume media changes every second day to prepare for bioprinting (Muñoz et al. 2020).

### Preparation of bioink

The bioink was prepared as previously described by Abelseth et al. (2018). For the bioink formulation, fibrinogen (cat no. 341578, EMD Millipore, Burlington, MA, USA) was prepared at a concentration of 20 mg/mL, 0.5% w/v sodium alginate (cat no. 180947, Sigma-Aldrich, St. Louis, MO, USA), and 0.3 mg/mL genipin (cat no. G4796, Sigma-Aldrich, St. Louis, MO, USA). It was then crosslinked with a mixture composed of 20 mg/mL CaCl2 (cat no. C1016, SigmaAldrich, St. Louis, MO, USA), 0.075% w/v chitosan (cat no. C3646, Sigma-Aldrich, St. Louis, MO, USA), and 1.7 U/mL thrombin (cat no. T7009, Sigma-Aldrich, St. Louis, MO, USA). Tris-buffered saline (TBS) was used as a buffer. The bioink formulation was sterilized using a 0.2 µm filter and was mixed with the primed neural progenitor cells (NPCs) at a concentration of 1 × 10^6^ cells/mL of bioink. The puro microspheres were sterilized with a plasma cleaner (Harrick Plasma, cat no. PDC-32G) and then added to the bioink at a concentration of 1 mg/mL.

### Bioprinting

Bioprinting was performed on the Aspect RX1 bioprinter (Aspect Biosystems) under sterile conditions in a biosafety cabinet. A DUO™ Printhead was used with the bioink connected to the Material 1 channel, and the crosslinker and buffer both connected to their respective channels. The pressures were set at 100 mbar, 90 mbar, and 500 mbar for the crosslinker, bioink, and buffer, respectively. Dome-shaped constructs were printed with final dimensions of approximately 10 mm in diameter and centre height of approximately 5 mm. A printing speed of 25 mm/s and a 40% rectilinear infill pattern were used to print the 9 layers that made up the construct (Fig. 1). After printing the constructs were transferred into a 12-well cell culture plate coated with PLO/laminin and containing BrainPhys™ Neuronal Medium (STEMCELL Technologies, cat no. 05790) supplemented with 100 ng/mL puro, 100 ng/mL FGF-8, 10 ng/mL BMP9, 100 ng/mL nerve growth factor (NGF) (Peprotech, cat no. 450–01), and 0.5% Penicillin-Streptomycin.

### Culture of bioprinted constructs

After bioprinting, constructs were cultured at 37 °C and 5% CO_2_ for up to 45 days. On days 1 and 3 the constructs were cultured in BrainPhys™ supplemented with 100 ng/mL puro, 100 ng/mL FGF-8, 10 ng/mL BMP9, and 100 ng/mL NGF. A half volume media change was performed on day 5 with BrainPhys™ supplemented with 100 ng/mL NGF and 5 ng/mL brain derived neurotrophic factor (BDNF) (Miltenyi Biotech, cat no. 130-093-811). On day 7, a full volume media change was performed with BrainPhys™ supplemented with 100 ng/mL NGF and 5 ng/mL BDNF. Half volume media changes were then performed every 2 days until day 45 with BrainPhys™ supplemented with 100 ng/mL NGF and 5 ng/mL BDNF (Fig. 1). Penicillin-Streptomycin was added to the media at a concentration of 0.5% to prevent contamination of the long-term culture.

### Cell viability

Cell viability was determined by using the LIVE/DEADTM Viability/Cytotoxicity kit (cat. No. L3224, Thermo Fisher, Waltham, Ma, USA). Cell viability was measured after bioprinting on days 1, 30, and 45. The media was removed from the constructs and washed twice with Dulbecco’s phosphate-buffered saline (DPBS). A solution composed of DPBS, 0.2% ethidium homodimer-1, and 0.05% calcein-AM was made up during the last wash. The solution was added to fully cover the constructs and then incubated for 45 min at 37 °C and 5% CO_2_. The constructs were imaged on a Leica DMI300 B microscope with an X-Cite Series 120Q fluorescent 25 light source (Excelitas Technologies) using an excitation of 488 nm to view the live cells and an excitation of 543 nm to view the dead cells. Images were obtained using a MicroManager imager (Crompton et al. 2013). Viability was calculated by taking images taken from one spot on the construct throughout the z-plane. They were combined using ImageJ V1.52a to represent the viability throughout the depth of the construct. The live and dead cells were then counted separately using ImageJ V1.52a software to calculate their percent viability for each day measured.

### Immunocytochemistry

Constructs were fixed on day 30 for immunocytochemistry (ICC) analysis. First the media was removed from the constructs, washed with phosphate buffer solution (PBS), and fixed with 0.5% paraformaldehyde (PFA) (cat no. FB002, Invitrogen). The constructs were incubated for 10 min at room temperature before the PFA was removed and the constructs were washed three times with PBS. After fixing, the constructs were manually sectioned in approximately 200 µm layers and placed on glass coverslips in PBS for staining. Each slice was permeabilized with 0.1% Triton-X (cat no. HT501128, Sigma, St. Louis, MO, USA), incubated for 10 min at room temperature followed by two washes with PBS. 5% normal goat serum (NGS) (cat no. ab7481, Abcam, Cambridge, UK) was added to each slice, incubated for 1 h at room temperature, then washed with PBS. The primary antibodies were diluted in PBS and added at different concentrations depending on previous literature and the manufacturers recommendation: recombinant Anti-FOXG1 (1:500), recombinant Anti-Choline Acetyltransferase (1:100), Beta 26 Amyloid Polyclonal (1:200), Phospho-Tau (Ser202, Thr205) Monoclonal (AT8), and anti-beta III tubulin, clone Tuj1 (1:1000) and then incubated at 4 °C overnight (Table 1). After incubation, the slices were washed three times with PBS with 5-min incubations at room temperature between each wash. The secondary antibodies: Goat anti-mouse (Alexa Fluor 488) (1:1000) and Goat anti-rabbit (Alexa Fluor 568) (1:1000) were diluted in PBS, added to the tissue slices and incubated for 1 h at room temperature in the dark. After incubation, the slices again were washed three times with PBS with 5-min incubations at room temperature between washes. 300 nM of DAPI (cat no. D1306, ThermoFisher, Waltham, MA, USA), diluted in PBS, was added to the slices, incubated for 5 min at room temperature with light protection, and then washed twice with PBS before imaging. The tissue slices were imaged on a FIPS-Zeiss Confocal Laser scanning microscope using a 63 × oil/water immersion lens and the images analysed using ZEN 3.5 (ZEN lite) blue edition software. Images were quantified in ImageJ V1.52a by subtracting the background fluorescence, adjusting the threshold until only the cells were in view, and then measuring the total area of each ICC marker in the image limited to the threshold view. The area for each marker and construct was then normalized over the area of DAPI to account for the different number of cells in each image.

**Table 1:** Antibodies used for immunocytochemistry staining

**Antibody**	**Raised In**	**Dilution**	**Company, Cat #**
FoxG1	Rabbit	1:500	Abcam, ab196868
ChAT	Rabbit	1:100	Abcam, ab181023
Amyloid beta	Rabbit	1:200	ThermoFisher, 71-5800
Tau	Mouse	1:500	ThermoFisher, MN1020
Anti-beta III Tubulin antibody	Rabbit	1:1000	Abcam, ab18207
Anti-beta-tubulin III antibody, Clone Tuj1	Mouse	1:1000	STEMCELL, 60052
Goat anti-mouse (Alexa Fluor 488)	Goat	1:1000	Invitrogen, A11029
Goat anti-rabbit (Alexa Fluor 568)	Goat	1:1000	Invitrogen, A11011

### Electrophysiology

Electrophysiological activity of the constructs was determined by adding Fluorescence Imaging Plate Reader Blue dye (FLIPR) Membrane Potential Assay Kit Blue (cat no. R8042, Molecular Devices, San Hose, Ca, USA) dye to the constructs at a ratio of 1:1 with the cell media (i.e. 500 µL cell media and 500 µL FLIPR Blue dye). This process was performed in a dark biosafety cabinet to avoid activating the dye. FLIPR blue dye was also added to constructs without cells and constructs containing microspheres without cells to determine the background fluorescence. The constructs were incubated for 30 min at 37 °C and 5% CO_2_ and then analysed on the TECAN infinite M200 Pro microplate reader to determine their baseline fluorescence. The microplate reader had a fluorescence excitation of 530 nm, fluorescence emission of 565 nm, and took 25 readings of each construct in a 5 × 5 square pattern. Stimulant dissolved in 500 µL dH_2_O was combined with 500 µL FLIPR Blue dye to obtain a final concentration of 56 mM potassium chloride (KCL) (cat no. P9541, Sigma) and 100 µM acetylcholine (cat no. A2661, Sigma). The constructs were again incubated for 30 min at 37 °C and 5% CO_2_ and the excitation was read on the microplate reader at the same settings as previously detailed. The change in fluorescence was calculated from Eq. 1 where F is the average fluorescence and F_0_ is the average background reading as outlined in the protocol by Robinson et al. (2019). The change in membrane potential was calculated from Eq. 2 where R is the gas constant, T is the average temperature, z’ is the apparent charge of the external dye concentration, and F is Faraday’s constant.

1 $$\Delta F= \frac{F- {F}_{0}}{{F}_{0}}$$

2 $$\Delta E= \frac{R \times T}{{z}^{^{\prime}}\times F} \times \mathit{ln}\left(\frac{1}{\frac{\Delta F}{{F}_{0}}+1}\right)$$

### Statistical analysis

Statistical analysis was completed using GraphPad Prism 5 statistical software. For electrophysiology experiments comparing at rest and excited readings, statistics were completed with a one-way paired student t-test with *p* < 0.05 (95% confidence level). A one-way student t-test was conducted to determine the statistical significance between the control and experiment groups for cell viability results and the comparison of the day 30 and day 45 resting cell membrane potential with *p* < 0.05 (95% confidence level). All results are given as the mean values ± standard deviation with a biological *n* = 3. For ICC analysis a 2-way ANOVA was run with Bonferroni multiple comparisons between all construct groups and markers with *p* < 0.05 (95% confidence level). All results are given as the mean values ± standard deviation with a biological *n* = 3.

## Results

### Microsphere characterization

**Fig. 2:**
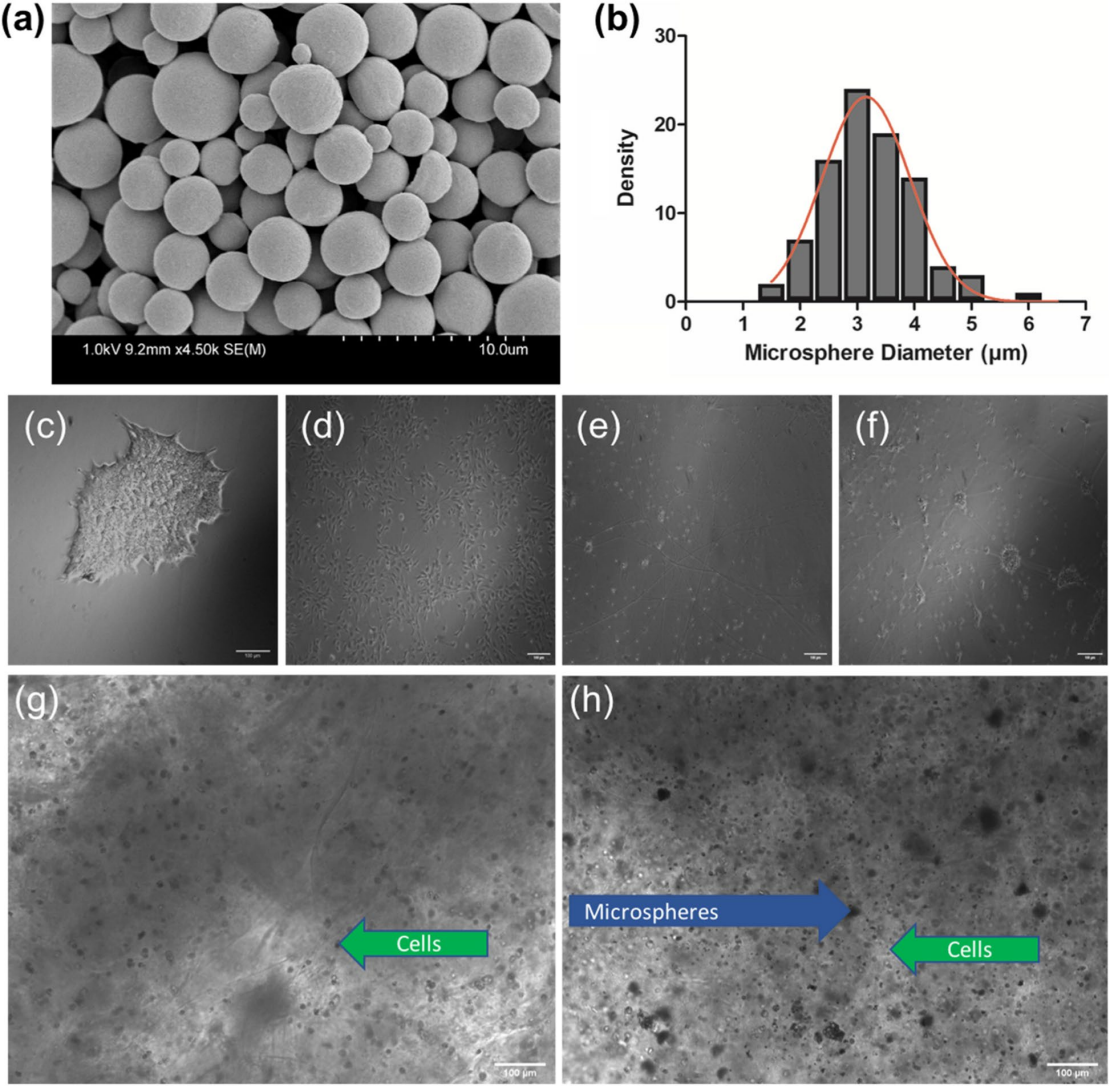
Characterization of PCL puro encapsulated microspheres. **A** SEM image demonstrating the size and morphology of the lyophilized microspheres. An accelerated voltage of 1.0 kV and working distance of 9.2 mm was used to acquire the image. Scale bar is 10.0 µm. **B** Histogram of puro microsphere diameters with a sample size of *n* = 90. Phase contrast images of **C** AD hiPSCs, **D** AD NPCs, **E** 2D cultured BFCN’s on day 10 and **F** day 20 of culture. Bright phase images of constructs with cells (**G**) and cells and microspheres (**H**). Scale bar is 100 µm

Puro encapsulated microspheres, 0.93 µg/mg (w/w puro/PCL), were created using an oil/water single emulsion process and characterized with SEM to evaluate their surface morphology and size distribution (Fig. 2a, b). The lyophilized microspheres were spherical with a smooth surface and a consistent diameter of 3.20 ± 0.82 µm, *n* = 90. The encapsulation efficiency of the microspheres was determined using HPLC. A standard curve was created based on five different dilutions of puro into ©. The encapsulation of puro in the puro/PCL microspheres was determined to be 76.4 ± 4.2% of the total amount of puro added during the oil/water emulsion. This was found by calculating the area under the peak and then comparing to the standard curve created. The PI of the microspheres was found using DLS to be 1 ± 0, *n* = 213. This indicates a highly polydisperse sample with a large size distribution of microspheres (Danaei et al. 2018).

### Bioprinted constructs

The cells were bioprinted in the NPC stage after differentiation from hiPSCs. After printing, they were directed to mature into BFCNs using the addition of small molecule growth factors to the media. This progression can be seen in the 2D controls in (Fig. 2©,d,©). By day 10 in the 2D controls, numerous neurite extensions had formed and on day 20 small neurospheres were observed with extensions between them. Constructs were bioprinted using the Aspect RX1 bioprinter (Fig. 2g, h). Dome-shaped constructs were created with a 10 mm diameter and approximate height of 5 mm at their highest point. Four groups of constructs were printed: healthy (HN1) and diseased (AD) models with cells and microspheres (CM) and healthy (HN1) and diseased (AD) models with only cells © (Table 2). The cells were added at a concentration of 1 million cells/mL of bioink and the microspheres at 0.5 mg/mL of bioink.

**Table 2:** Description and acronyms of the bioprinted groups

Description	Acronym
Bioprinted HN1 NPCs	HN1 C
Bioprinted HN1 NPCs with microspheres	HN1 CM
Bioprinted AD NPCs	AD C
Bioprinted AD NPCs with microspheres	AD CM

### Cell viability

**Fig. 3:**
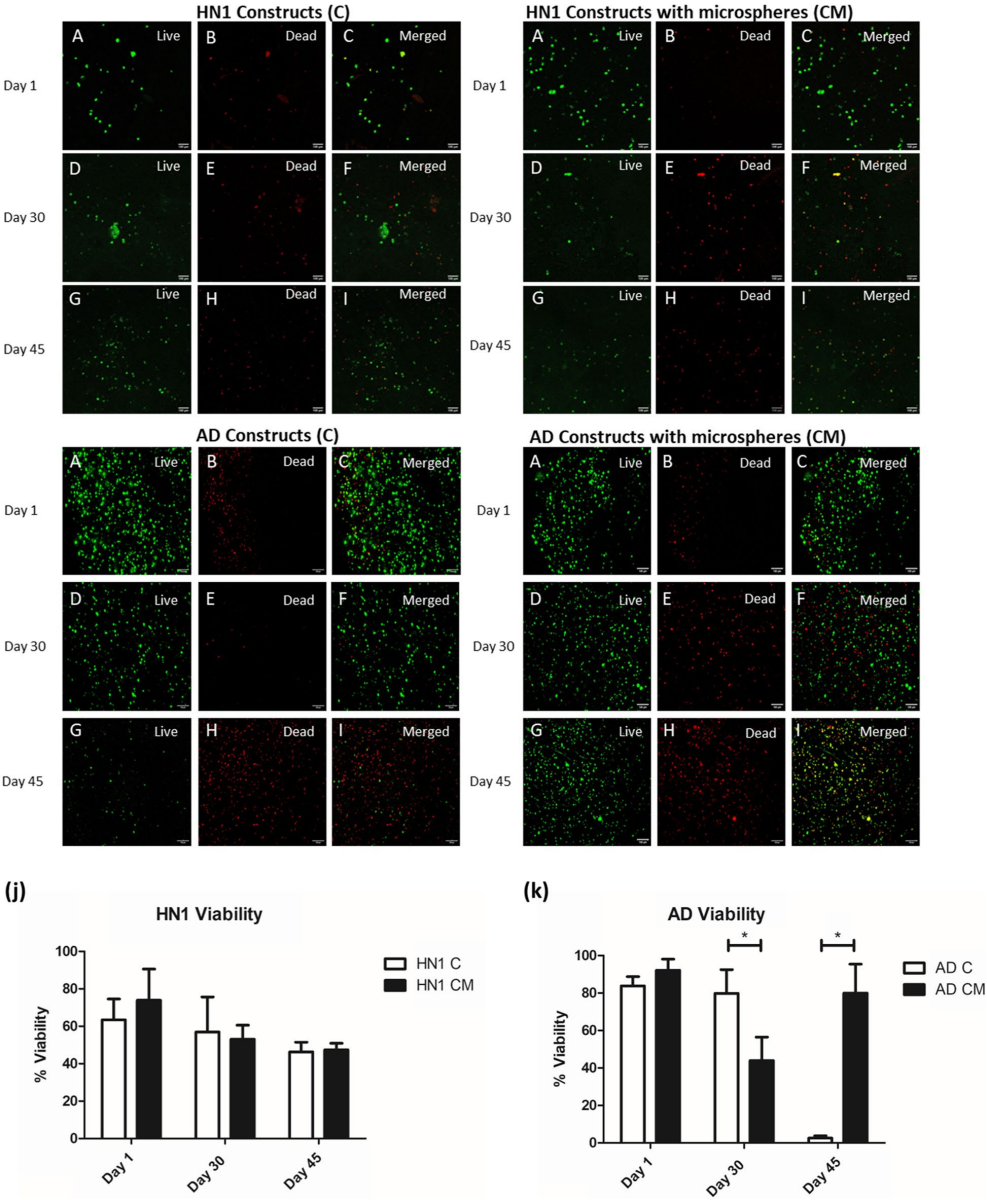
Cell viability images, live (A, D, G), dead (B, F, H), and composite (C, F, I) of HN1 bioprinted constructs, HN1 bioprinted constructs containing puro releasing microspheres, AD bioprinted constructs, and AD bioprinted constructs containing puro releasing microspheres on days 1,30, and 45 of culture. Scale bar is 100 µm. A comparison of the cell viability for (j) HN1 cells and (k) AD cells on days 1, 30, and 45 between constructs with (CM) and without © microspheres. All groups *n* = 3 constructs

Four groups were bioprinted: HN1 and AD NPCs treated with small molecules and HN1 and AD NPCs with PCL loaded-puro microspheres treated with small molecules. Cell viability was quantified on days 1, 30, and 45 for all bioprinted groups (Fig. 3). For the healthy controls, on day 1 the cell viability for the HN1 CM group was 73.86 ± 16.68% higher than the HN1 C group of 63.35 ± 11.26% although no statistical significance was observed. On day 30 the viability decreased slightly for each group with a viability of 56.90 ± 18.73% for the HN1 C group and 53.04 ± 7.54% for the HN1 CM. Finally, on day 45 both the HN1 C and CM groups had a similar viability of 46.26 ± 5.18% and 47.39 ± 3.51% respectively. On day 1 directly after bioprinting, cell viability for the AD C group was 83.78 ± 4.96% while AD CM had a viability of 92.16 ± 5.94%. On day 30, cell viability decreased for both groups of constructs with the AD C group having a viability of 78.81 ± 12.63% and the AD CM group with a viability of 43.92 ± 12.52%. Finally, the biggest disparity between groups was seen on day 45 with the AD C group having a viability of 2.58 ± 1.17% and the AD CM group 79.96 ± 15.44% viable.

### Immunocytochemistry

**Fig. 4:**
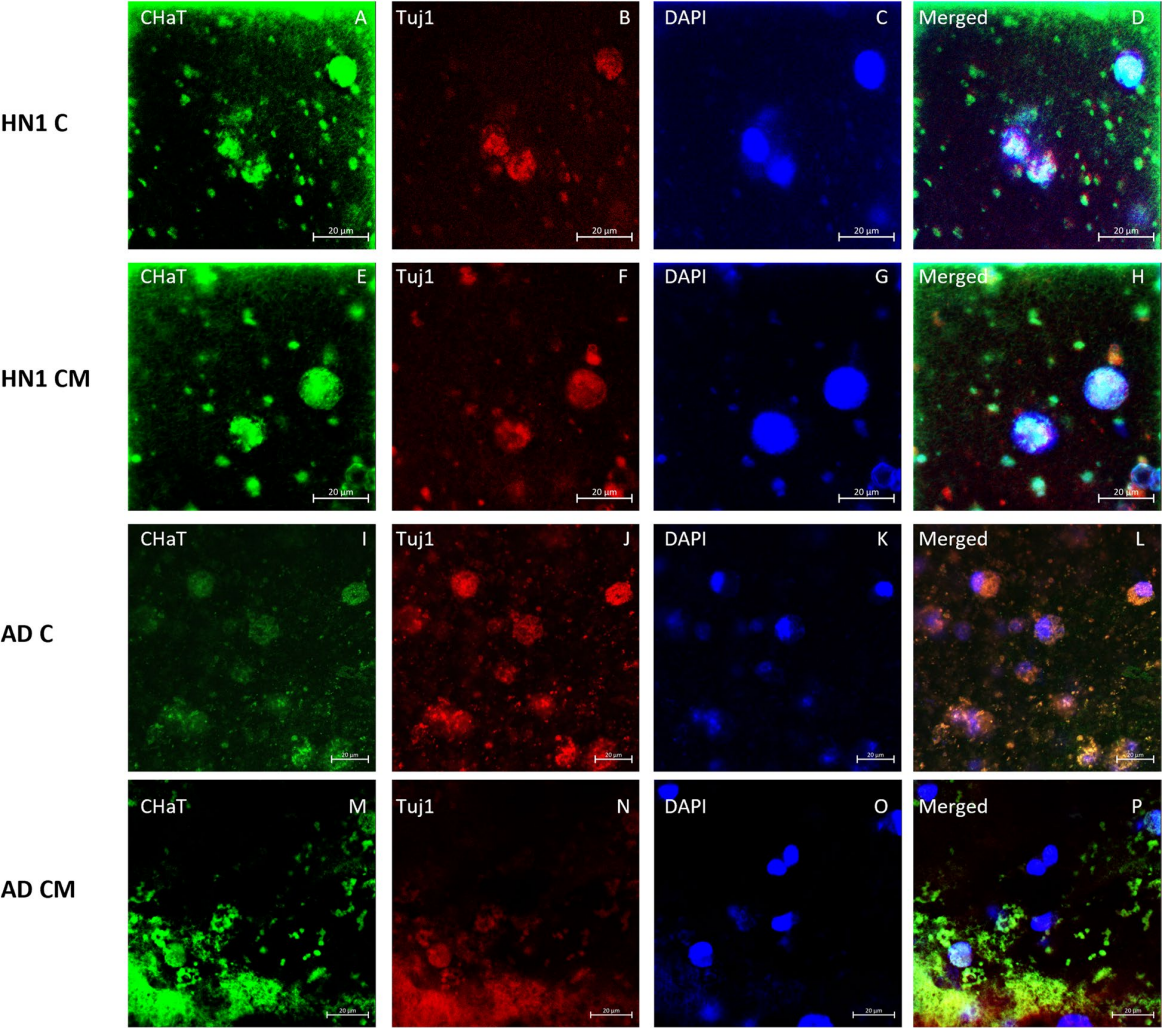
Immunocytochemistry analysis of HN1 and AD bioprinted constructs on day 30 of culture. Successful generation of BFCN models are indicated by the cholinergic marker ChAT and neuronal marker Tuj1. Scale bar is 20 µm

**Fig. 5:**
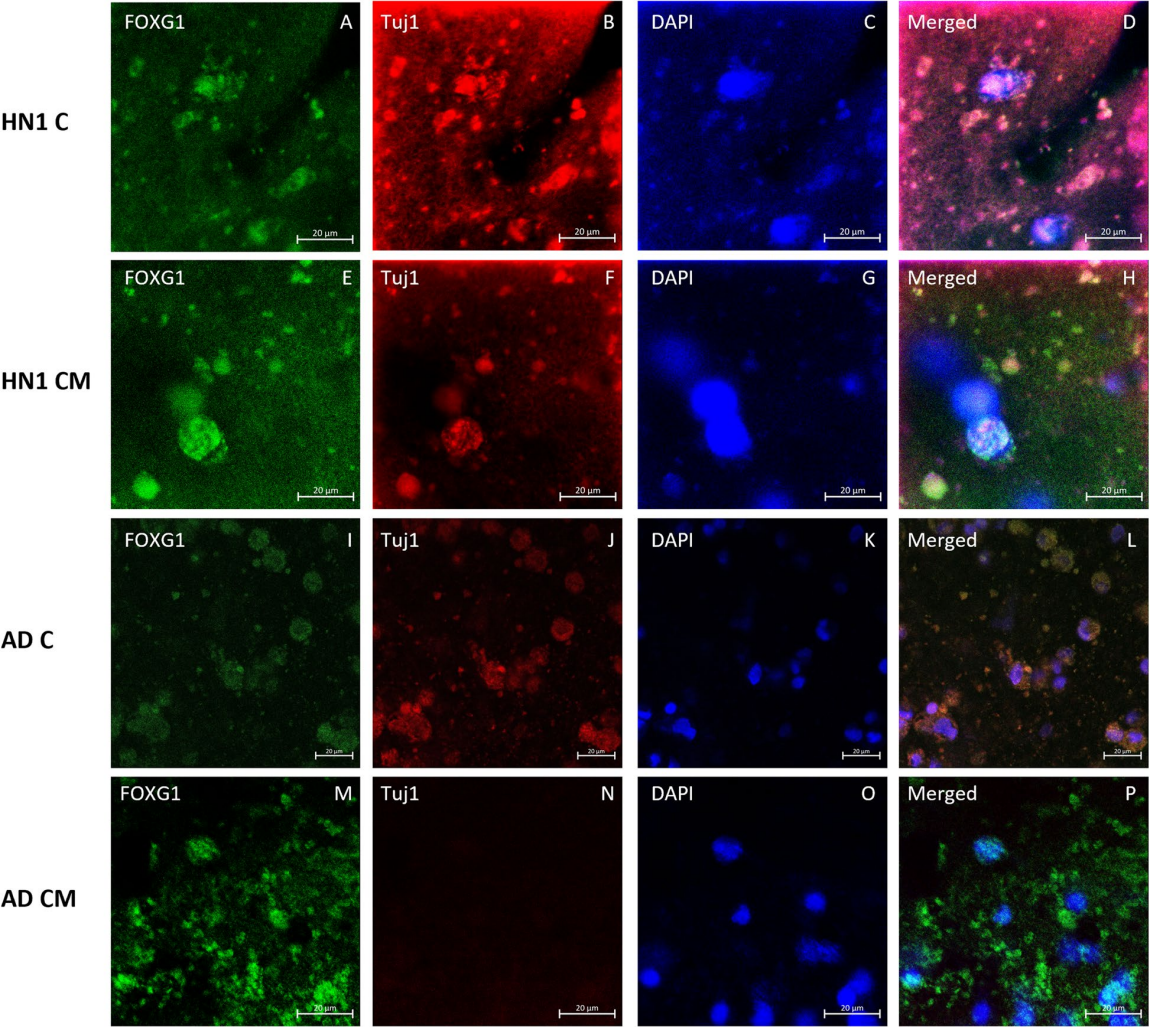
Immunocytochemistry analysis of HN1 and AD bioprinted constructs on day 30 of culture. Successful generation of BFCN models are indicated by the basal forebrain marker FOXG1 and neuronal marker Tuj1. Scale bar is 20 µm

**Fig. 6:**
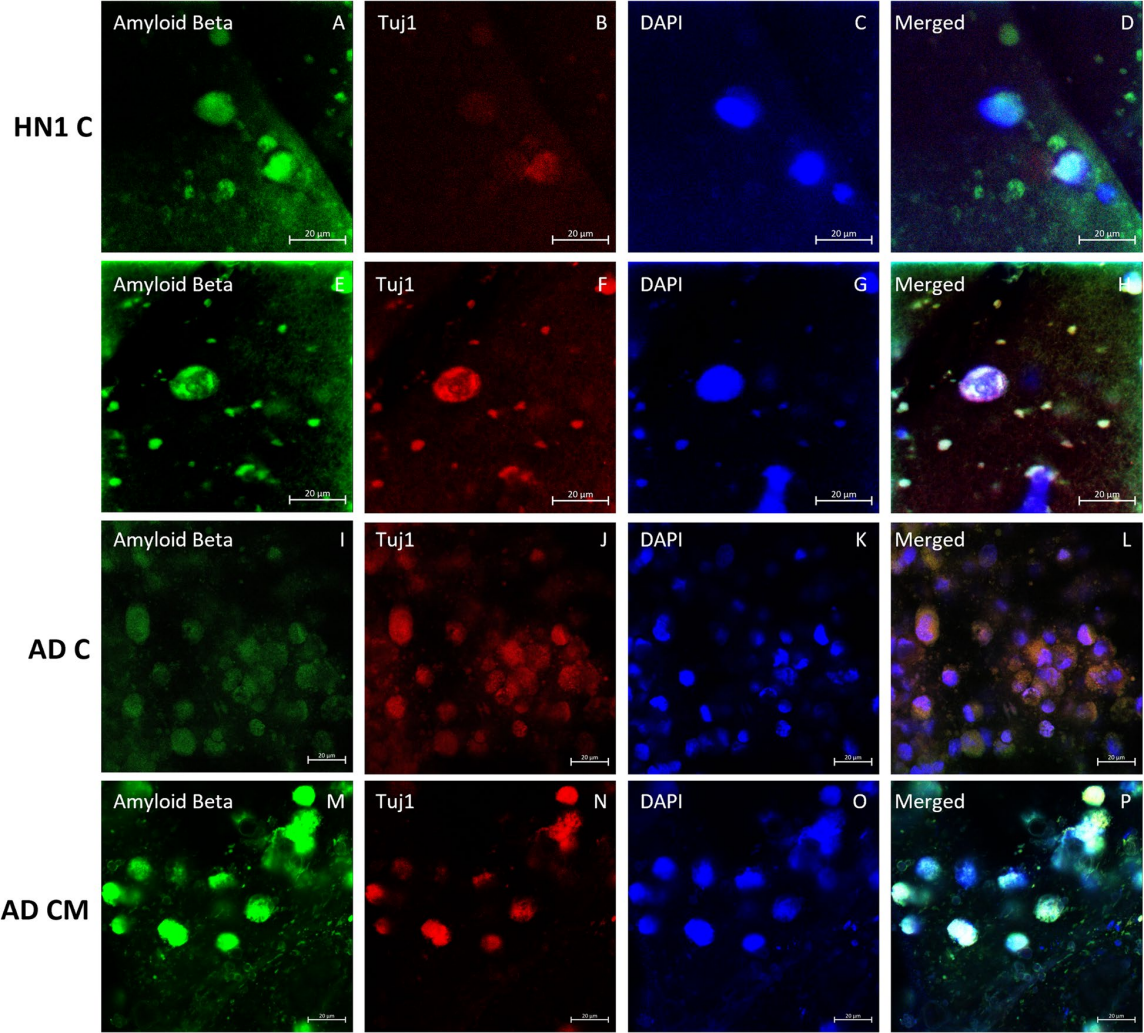
Immunocytochemistry analysis of HN1 and AD bioprinted constructs on day 30 of culture. The marker of AD, amyloid beta, is shown along with the neuronal marker Tuj1. Scale bar is 20 µm

**Fig. 7:**
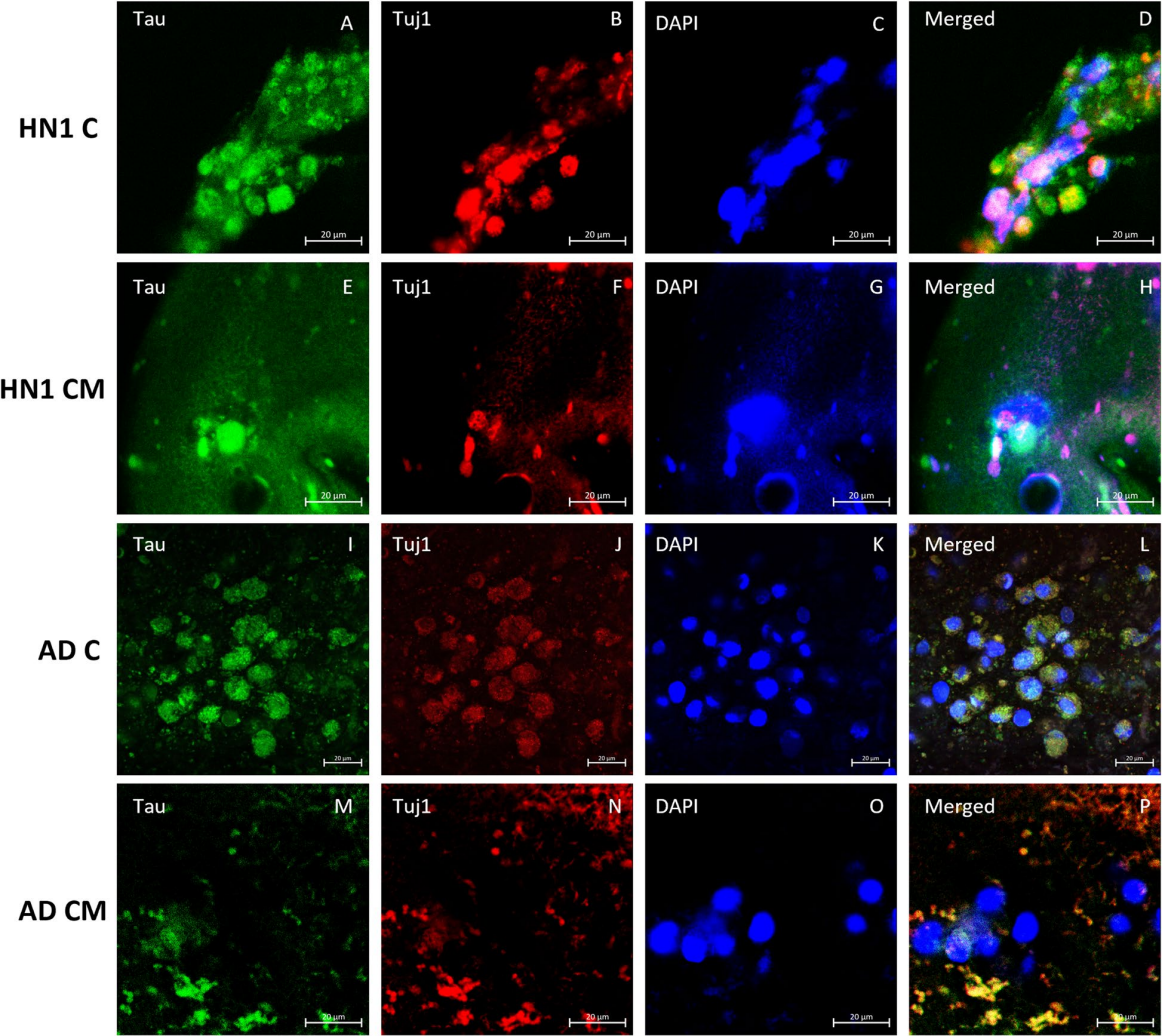
Immunocytochemistry analysis of HN1 and AD bioprinted constructs on day 30 of culture. The marker of AD, tau, is shown along with the neuronal marker Tuj1. Scale bar is 20 µm

**Fig. 8:**
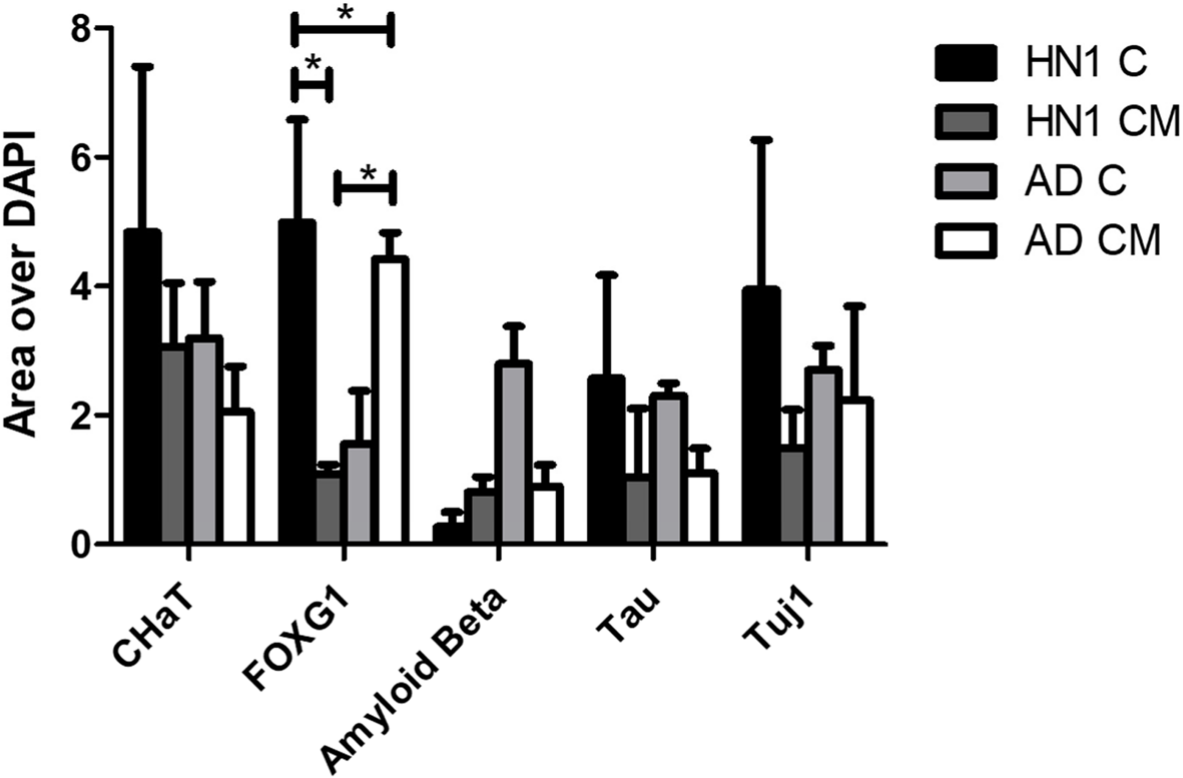
The qualitative results for the area of each marker normalized over the area of DAPI is shown. A 2-way ANOVA was run with Bonferroni multiple comparisons between all construct groups and markers with *p* < 0.05 (95% confidence level) to determine statistical significance, *n* = 3 for all groups

ICC analysis was performed on all groups of bioprinted constructs on day 30 of culture. All constructs were stained with the neuronal marker β-tubulin III (Tuj1), the basal forebrain marker FOXG1, and cholinergic enzyme choline acetyl transferase (ChAT) to verify the presence of BFCN’s. Amyloid beta, the major component of amyloid plaques in AD patients’ brains, and tau, the major component of neurofibrillary tangles in AD patients’ brains, were stained and observed in the cultures. Finally, the nucleic acid stain DAPI was added to determine the number of nucleated cells in each image. Stains of the markers were all seen throughout the different cultures (Figs. 4, 5, 6, 7 and 8).

### Electrophysiology

**Fig. 9:**
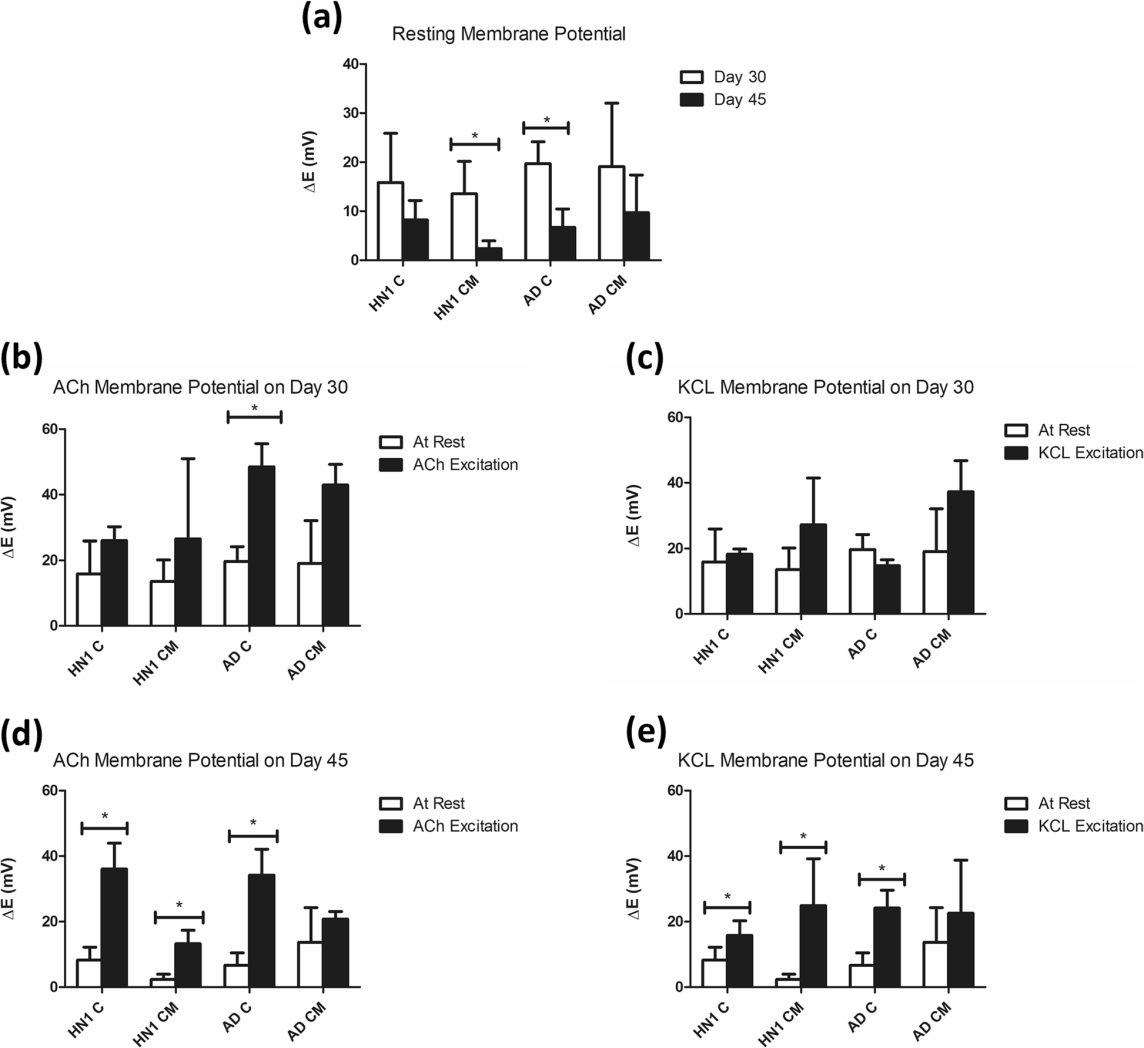
**a** Microplate readings of HN1 and AD constructs with microspheres (CM) and constructs (C) all at rest. All groups *n* = 3 and statistics completed with one-way student t-test, * shows significance between resting membrane potential on days 30 and 45. Microplate readings of all bioprinted constructs membrane potentials measured at rest and excited with Ach on day 30 (**b**) and day 45 (**c**) of culture. And bioprinted constructs membrane potentials measured at rest and excited with KCL on day 30 (**d**) and day 45 (**e**) of culture. All groups of *n* = 3 and statistics completed with one-way paired student t-test, * shows significance between at rest and excited readings

Electrophysiology analysis was performed on all groups of bioprinted constructs on days 30 and 45 of culture by measuring changes in fluorescence that were then calculated to indicate a corresponding membrane potential. The resting membrane potential of the constructs was compared on days 30 and 45 of culture to evaluate their increase in electrophysiological maturity. All groups saw a decrease in resting membrane potential from day 30 to day 45 with the lowest membrane potential observed in the HN1 CM group of 2.34 ± 1.61 mV on day 45 compared to 13.54 ± 6.60 mV on day 30 indicating that the HN1 cells became the most electro-physically mature (Fig. 9). All groups were excited with both KCl and ACh on days 30 and 45 of culture. After excitation with KCL on day 30 no statistically significant increases in membrane potential were seen due to large standard deviations. By day 45 all groups showed an increase in membrane potential after KCL excitation with again the HN1 CM constructs showing the highest excitation of 24.86 ± 14.33 mV (Fig. 8). After excitation with ACh on day 30, all groups showed an increase in membrane potential with the AD groups having the largest response. By day 45 statistically significant increases in potential were shown in all groups other than AD CM with both HN1 C and AD C having a larger increase compared to both CM groups. HN1 C showed the largest increase in membrane potential with an excitation of 36.05 ± 7.86 mV.

## Discussion

In this study, patient-derived hiPSCs were differentiated into NPCs and bioprinted with a bioink that incorporated microspheres to direct their maturation into cholinergic neurons. Puro is a hydrophobic small molecule that can function as a sonic hedgehog (SHH) agonist and was encapsulated into the microspheres. It was chosen for this project because it has been shown to generate neural progenitor cells that express the transcription factor ISLET1 (ISL1), a specific BFCN marker that is required for its forebrain fate (De la Vega et al. 2018; Muñoz et al. 2020). In previous studies, the generation of BFCNs from hiPSCs was accomplished with the addition of SHH, however, Hu et al. substituted puro and found it to also be effective in deriving BFCNs (Bissonnette et al. 2011; Crompton et al. 2013; Liu et al. 2013; Hu et al. 2016). The substitution of puro is advantageous when incorporating small molecules into microspheres; it is less sensitive than SHH and its risk of denaturing during the o/w emulsion process is greatly reduced. The small size of microspheres (3.20 ± 0.82 µm, *n* = 90) was ideal for this study as the microspheres were incorporated into bioink that was extruded through a microfluidic printhead. The small size and smooth surface morphology worked together to help minimize the amount of clogging that occurred in the microfluidic printhead channels. Although the small size of microspheres was ideal for bioprinting, the high PI (1 ± 0) indicates a polydisperse sample of microspheres with a large size distribution. This can most likely be attributed to the o/w emulsion method of fabrication. The use of microfluidics in the future for fabrication could help with creating a more monodisperse population (Forigua et al. 2022). Increased cell viability is often found when growth factor microspheres are incorporated into bioinks (Tan et al. 2016). Royce et al. showed that a combined fibrin-microsphere system caused the encapsulated growth factors to be delivered over a longer period of time compared to constructs with microspheres alone, and that over 48 h fibroblast proliferation was increased when growth factors were encapsulated in the microspheres (Royce et al. 2004). The encapsulation efficiency of the microspheres used in this project was determined to be 76.42 ± 4.23% which is slightly lower than previously published results of 84 ± 2.12% (De la Vega et al. 2018). This could be due to how the drug is extracted for testing, allowing for the possibility that not all the PCL was fully dissolved in the ACN. The higher encapsulation efficiency compared to other small molecule encapsulated microspheres created with the same method (i.e., guggulsterone, retinoic acid) is most likely a result of the organic solvent solubility of puro (Gomez et al. 2015). De la Vega et al. showed that there was an initial 16% drug release of puro on day 1. Over the next 45 days, 91 ± 1.7% of the encapsulated puro was released in a slow, linear fashion (De la Vega et al. 2021). Gomez et al. showed that an increase in the amount of drug encapsulated will cause a greater cumulative release in a shorter amount of time (Gomez et al. 2015). This could be used in future studies to further customize the release rates and therefore, the directed differentiation of various cells. The puro microspheres were added to the bioink in this project to help regulate the cell division and differentiation of the BFCNs without any toxicity being introduced during the degradation of the PCL.

Cellular viability was evaluated at different time points to assess how the cells were proliferating and surviving in the bioink as well as to determine whether the addition of microspheres had a positive effect on the cells and to observe the difference between the healthy and diseased populations. Bioprinting can stress the cells as they are exposed to shear stress during extrusion printing. The bioink developed by Abelseth et al. has a low viscosity and can be printed and crosslinked with the LOP microfluidic printhead at a low pressure minimizing the stress on the cells (Abelseth et al. 2018). The natural biomaterials that compose the bioink, including the fibrin, alginate, and chitosan also contribute to cellular survival as well as promoting the proliferation and differentiation of NPC’s into mature neurons (De la Vega et al. 2021; Benwood et al. 2021). The genipin, calcium chloride, and thrombin components work to crosslink the bioink and create the desired mechanical properties for the cells (De la Vega et al. 2021; Sharma et al. 2021a). Directly after printing, the cells in the AD C group showed a viability over 83% while the AD CM constructs viability was over 92%. A similar trend was shown with the HN1 C and CM groups. Although they both showed a lower viability compared to the AD constructs the HN1 CM group had a viability over 73% and the HN1 C had a viability of 63%. Although there was no statistical significance between the two groups, the slight increase in viability for the constructs with microspheres could be a result of the microspheres providing a protective environment around the cells, therefore helping to reduce any damage that could occur during the printing process. The slight increase in viability of the CM groups on day 1 could also be a result of the initial burst release of puro from the microspheres leading to increased cellular proliferation. On day 30, both HN1 groups had decreased slightly with similar viability levels of around 55%. The AD groups however showed a significant difference with the AD C group having a viability of 78.81 ± 12.63% greater than the CM group of 43.92 ± 12.52%. However, by day 45, the AD CM group was significantly healthier than the C group with 77% more live cells. The increase in viability from day 30 to 45 in the AD CM constructs could be a result of the cells going through apoptosis and triggering proliferation (Guerin et al. 2021). Sharma et al. show that the addition of microspheres to the bioink increased the mechanical strength of the constructs as well as their stability (Sharma et al. 2021a). The mechanical properties from the addition of microspheres as well as the slow release of puro could have aided in the proliferation and survival of the cells in the AD CM constructs. In contrast, the HN1 groups showed a slight decrease in viability from day 30 and both had a cell viability of just under 50% on day 45 of the culture. In comparison to a 2D culture, where the dead cells float to the surface and are removed from the culture with a media change, the tissue constructs are unable to remove the dead cells. The 3D system did not display any neurite extensions unlike the 2D culture. The lack of visualization of neurite extensions in the constructs could be a result of the slicing and permeabilization and fixation required to prepare the tissues for ICC. The tissue constructs may better replicate the environment of the human brain as the 2D culture only provides side contact with other cells against a hard, flat surface, no cell-ECM interaction, and does not require nutrient/oxygen diffusion dynamics (Centeno et al. 2018). Finally, high standard deviations were observed when evaluating the cellular viability as well as visually observing different sections of a single tissue construct. This is likely a result of the bioprinting process where cells can clump together and settle in the bioink throughout printing. Future work should focus on ensuring that cells are distributed more evenly throughout the tissue and determining the cell density throughout the entire construct after long term culture.

ICC was done on the constructs for two main purposes: i) to confirm the presence of BFCN’s with the staining of Tuj1, FOXG1, and ChAT; and ii) to see if the AD models displayed the two hallmarks of AD, namely amyloid plaques and hyperphosphorylated tau. BFCNs were the neurons chosen for this study because they are critical for the regulation of brain function and their dysfunction occurs early in the progression of AD. Several protocols have been published showing the differentiation of hiPSCs to BFCN’s (Bissonnette et al. 2011; Crompton et al. 2013; Liu et al. 2013). For this study, the protocol for differentiation was based off the work done by Munoz et al. using a combination of small molecules, specifically puro, FGF8, BMP9, NGF, and BDNF, added at specific timepoints of culture (Muñoz et al. 2020). Puro was substituted for SHH in the current study and was added to induce SHH signaling. In vivo, SHH induces the ventralization of the neural tube where BFCNs form (Muñoz et al. 2020). FGF-8 has been shown to produce expression of FOXG1 in the developing telencephalon and was added to mimic its development (Muñoz et al. 2020; Crompton et al. 2013). Together the treatment of puro and FGF-8 worked to guide the differentiation of the NPCs to a forebrain progenitor fate (Bissonnette et al. 2011). BMP9 is briefly expressed in vivo in the septum during development and was included to help increase the cholinergic phenotypes of the cells (Bissonnette et al. 2011). For the maturation of NPCs to BFCNs, NGF and BDNF was added to the media to increase the survival and maturation of the cells, promote ChAT activity, and increase cholinergic differentiation (Alderson et al. 1990; Auld et al. 2001; Sofroniew et al. 2001). To confirm the maturation of BFCNs, the neuronal marker Tuj1 which is only found in the central and peripheral nervous system, was seen most consistently in day 30 images of both groups of constructs. As a member of the tubulin family, Tuj1 supports axon development and maintenance and occurs in the cytoplasm and cytoskeleton of cells (Engel et al. 2016). Next, FOXG1, a developmental marker of neurons in the developing telencephalon, has been found to be located directly outside of the nucleus of the majority of cells (Bissonnette et al. 2011). It occurred in all stained groups except for day 15 of the CM group. Finally, all groups were stained for ChAT, which is typically distributed in the cytoplasm. It is an enzyme that is expressed by BFCNs, and it synthesises acetylcholine (ACh) at cholinergic synapses. Previous 2D studies have shown that the co-expression of ChAT, FOXG1, and Tuj1 indicates the successful generation of BFCNs (Bissonnette et al. 2011; Muñoz et al. 2020; Hassan et al. 2018). By day 30 of culture, both groups had expressed these markers. A direct correlation between the activity of ChAT and its synthesis of acetylcholine with increased signs of AD has been observed (Pedersen et al. 1996). Hampel et al. found that in post-mortem brains of patients with AD, there were an increased number of neuritic plaques made up of amyloid beta, corresponding to a decrease in ChAT activity (Hampel et al. 2018). Amyloid beta peptides are 40–43 amino acids long and make up the neuritic plaques and neurofibrillary tangles that occur in AD. They are generated from the amyloid beta precursor protein (APP) in a two-step process: 1: cleavage of APP by beta-secretase (BACE) produces a cellular secretion of a segment of APP; 2: gamma secretase cleaves an intra-membrane site in the carboxyl terminal domain of APP which generates the amyloid beta peptide (Lee et al. 2016). Amyloid beta has also been found to accumulate in cognitively healthy individuals and was observed in both the healthy and diseased bioprinted cell models (Majdi et al. 2020). Imaging was obtained on day 30 and a longer-term culture could lead to the observation of increased amyloid plaque formation compared to the healthy controls. Finally, all groups were stained for the neuronal microtubule associated protein Tau. It is typically found on axons and in healthy cultures it works to stabilize microtubules as well as encourage tubulin polymerization. In AD however, when tau is hyperphosphorylated, the microtubule binding function of tau is compromised which leads to the destabilization of microtubules and the eventual degeneration of the AD neurons (Majdi et al. 2020). Tau is also one of the main parts of paired helical filaments (PHF) which creates neurofibrillary lesions in AD (Majdi et al. 2020). Phosphorylated tau has been found to accumulate early in the AD progression in BFCNs and has been shown to correlate with the cognitive decline of patients (Ma et al. 2020). Similar to the amyloid beta, no significant differences were observed between the healthy and diseased constructs and the group with microspheres and without. Again, a longer culture could lead to the visualization of hyperphosphorylated tau aggregates. Previous studies have shown the progression of amyloid beta and tau as the culture has lengthened (Arber et al. 2021).

The measurement of cell membrane potential can be used to validate the functionality of the neurons created (Robinson et al. 2019). Neurons are considered mature when they have a resting membrane potential of -70 mV compared to the more depolarized resting membranes of immature neurons. They are considered functional when their response to stimulation is the firing of action potentials. Conventionally, this would be measured by the use of patch clamping, where the changes in membrane potential due to the opening and closing of ion channels on the cell’s membrane is quantified (Crompton et al. 2013). This procedure is not possible on bioprinted constructs because it requires the neurons to be pierced with electrodes (Cahalan and Neher 1992). Robinson et al. developed a protocol that uses the voltage-sensitive fluorescent dye FLIPR Blue to measure the electrophysical activity of neurons in tissues (Robinson et al. 2019). The dye has a high signal-to-noise ratio and when membrane depolarization occurs, it will attach to the intracellular hydrophobic sites and fluorescence will be emitted. When hyperpolarization occurs, the opposite happens resulting in a decreased fluorescent emission. These processes can then be quantified on the microplate reader allowing for the detection of electrophysical activity of the neurons (Fairless et al. 2013). The resting membrane potential of all four groups (HN1 C, HN1 CM, AD C, and AD CM) were quantified on days 30 and 45. On day 30, the four groups had resting membrane potentials ranging from 13 – 19 mV. By day 45 all four groups had a resting potential under 10 mV, with the HN1 CM group having a membrane potential of 2.34 mV indicating that the addition of microspheres to the constructs increased the rate at which the cells became electro-physically mature (Restan Perez et al. 2021). In vivo, neurons will display a resting potential of -70 mV, which is far from the approximate 10 mV found in the current cultures. This indicates that only immature electrical activity is being observed and that the culture is not purely BFCNs. Gonzales et al. showed that electrical activity was not seen until after 90 days of culture again, indicating that only immature electrical activity is occurring here (Gonzalez et al. 2013). The cells were stimulated with KCl to induce a membrane potential. Excitation was observed in all groups on both day 30 and 45 except for the AD C constructs on day 30. Overall, when the BFCN constructs and cells were stimulated with KCL to induce a membrane potential, an increase in fluorescence above the baseline was observed. This indicates that both the disease and healthy models had functional voltage-gated channels, implying that the cells can function and send electrical signals to transmit information (Crompton et al. 2013). Duan et al. found that AD neurons also expressed functional voltage-gated calcium channels and after KCL stimulation the amount of calcium influx through the channels was not significantly different when compared to the healthy controls (Duan et al. 2014). In healthy cells, Crompton et al. demonstrated that the cholinergic agonist carbachol (CCH) and KCL showed increases of 47.5 ± 7.9% and 67.3 ± 8.1% fluorescence over the baseline level (Crompton et al. 2013). Along with functional voltage-gated channels, this would suggest that functional cholinergic receptors and a cholinergic phenotype are occurring within their cultures (Crompton et al. 2013). ACh is an excitatory neurotransmitter in the brain that is active throughout the basal forebrain, basal ganglia, and cortex (Hampel et al. 2018). Cholinergic signal transduction is correlated with memory, cognition, and learning is regulated by ACh; however, patients with AD will usually have a deficient amount of ACh, causing damage to the cholinergic signal transduction (Chen et al. 2022). All four groups were stimulated with ACh on days 30 and 45 and fluorescence levels above the baseline were observed, although due to large standard deviations not all showed significant results. The excitatory response after stimulation with Ach suggests the presence of cholinergic receptors. AD pathology has a complex effect on the electrical function and cholinergic neuromodulator role of BFCNs. Hypo and hyperactivity at the synapses have been reported and their synaptic dysfunction also correlates with the cognitive decline of patients (Crompton et al. 2013). ACh response is an interesting marker to investigate because healthy cognition, memory, and learning relies on cholinergic signal transduction, which in turn depends on ACh (Chen et al. 2022). Further work in electrophysiological analysis could include the addition of different neurotransmitters or ion blockers to the media. This would enable the evaluation of specific ion channel responses as well as increase the depth of understanding of what is occurring in the tissue model. As well, ACh-deficiency in AD patients has shown to lead to a decline in cognitive and behavioural function so evaluating the release of ACh could lead to important insights in understanding the progression of AD (Chen et al. 2022; Crompton et al. 2013). For this study, NGF and BDNF were added to the cell media to help direct the maturation of the BFCNs (Muñoz et al. 2020). Those two neurotrophic factors have also been found to become dysregulated in AD as well as create a loss of neuronal markers and shrinkage (Chen et al. 2022). Varying the amount of those two factors added to the cultures would determine if they had a positive or negative effect on the disease progression of the cells.

Future work on this model should focus on optimizing culture conditions and imaging techniques to be able to include nerve processes in the tissue construct as well as increase long term cellular viability. In future experiments this could be achieved by creating a larger model with vasculature to ensure that nutrients and oxygen are able to reach the centre of the tissue structure for cell survival. These larger models could include different co-cultures such as astrocytes or mesenchymal stem cells (MSCs) to explore their effect on the progression of the disease. Previous studies have shown that MSCs can increase the levels of ACh and BDNF which may have a positive effect on the function of BFCNs. Further studies have shown that grafted MSCs have reduced the amount of amyloid beta plaque deposits in AD brains (Chakari-Khiavi et al. 2019; Shin et al. 2014).

## Conclusions

In this study, AD neural tissue models have been successfully bioprinted using a fibrin-based bioink with a microfluidic-based extrusion printhead system. Patient-derived hiPSCs were successfully differentiated and matured into neurons that showed BFCN markers, one of the first cell types to be affected in the progression of AD. The successful incorporation of microspheres into the bioink allowed for increased cell viability of the constructs. The expressions of neuronal and BFCN markers Tuj1, ChAT, and FOXG1 were observed along with the AD markers amyloid beta and tau. Finally, immature electrical signalling was observed when the tissue constructs were exposed to KCL and ACh. These neural tissue constructs show potential in the use of patient-specific drug screening as well. The ability to compare and evaluate the cell viability, electrophysiology, as well as evaluating the presence of amyloid beta and tau all could be valuable when evaluating different treatment options. They also show potential as a model to increase the understanding of the progression of AD.

## Supplementary Information

**Additional file 1: Supplemental Figure 1.** ICC analysis of neural progenitor markers to confirm the successful neural induction of hiPSCs to NPCs.

## Data Availability

All relevant data is included in the manuscript and the supplementary materials.

## Abbreviations

ACh: Acetylcholine
ACN: Acetonitrile
AD: Alzheimer’s disease
APP: Amyloid precursor protein
BDNF: Brain derived neurotrophic factor
BFCN: Basal forebrain cholinergic neurons
BMP9: Bone morphogenic protein 9
ChAT: Choline acetyl transferase
CNS: Central nervous system
DCM: Dichloromethane
DLS: Dynamic light scattering
DPBS: Dulbecco’s phosphate-buffered saline
ECM: Extra cellular matrix
FAD: Familial Alzheimer’s disease
FGF-8: Fibroblast growth factor
FLIPR: Fluorescence Imaging Plate Reader Blue dye
FOXG1: Forkhead box G1
hiPSC: Human induced pluripotent stem cell
HPLC: High performance liquid chromatography
ICC: Immunocytochemistry
KCL: Potassium chloride
LOP: Lab on printer
MSC: Mesenchymal stem cell
NFT: Neurofibrillary tangle
NGF: Nerve growth factor
NIM: Neural induction medium
NMDA: N-methyl-D-aspartic acid
NPC: Neural progenitor cell
NPM: Neural progenitor medium
o/w: Oil/water
PBS: Phosphate buffered saline
PCL: Poly-caprolactone
PFA: Paraformaldehyde
PI: Polydispersity index
PTFE: Polytetrafluorethylene
Puro: Puromorphamine
PVA: Polyvinyl alcohol
RA: Retinoic acid
sAD: Sporadic Alzheimer’s disease
SEM: Scanning electron microscopy
SHH: Sonic hedgehog agonist
Tuj1: β-tubulin III
