# Supplementary material for: 3D bioprinting patient-derived induced pluripotent stem cell models of Alzheimer’s disease using a smart bioink
Source: Bioelectron Med. 2023 May 24;9:10. doi: 10.1186/s42234-023-00112-7 (PMC10207712; doi:10.1186/s42234-023-00112-7)
Supplement: Supplementary file 1 — Additional file 1: Supplemental Figure 1. ICC analysis of neural progenitor markers to confirm the successful neural induction of hiPSCs to NPCs. [file 42234_2023_112_MOESM1_ESM.docx]

**Supplemental Figure**

**
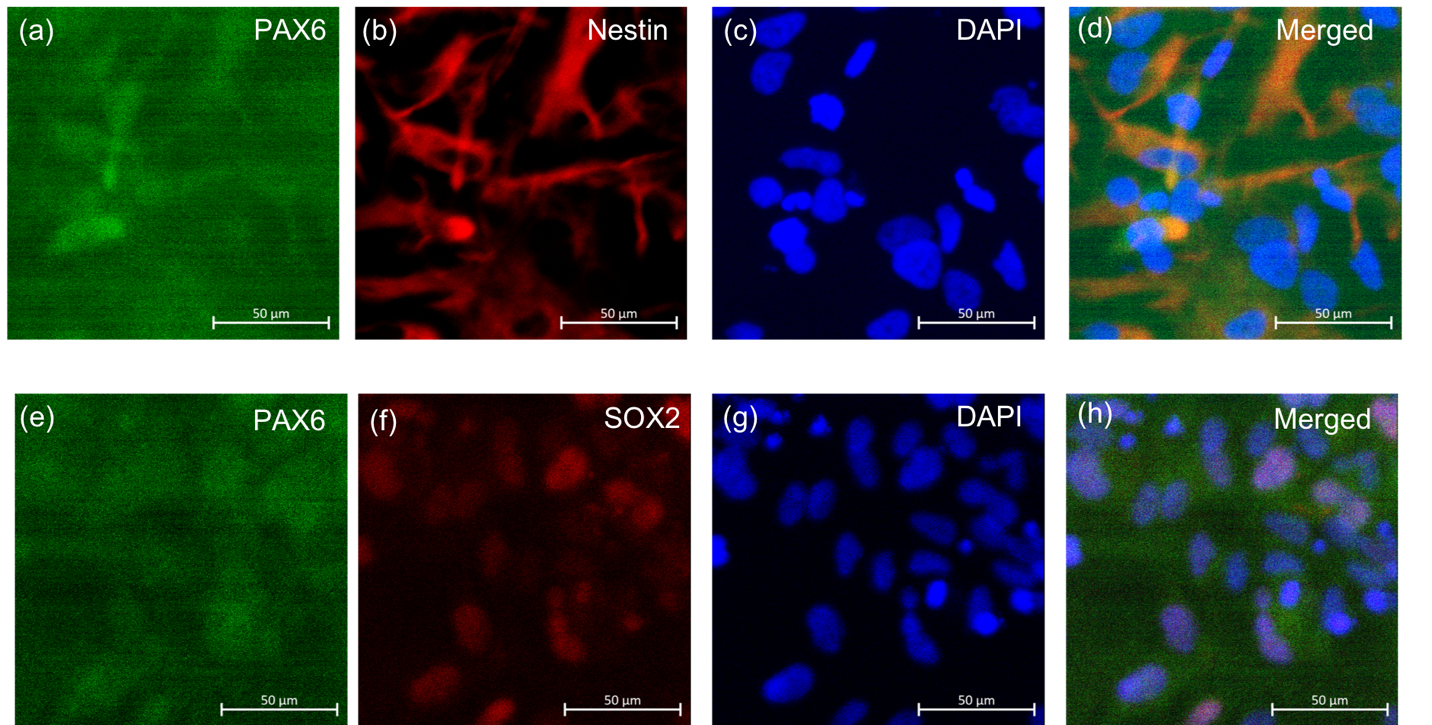
**

Supplemental Figure 1: ICC analysis of neural progenitor markers to confirm the successful neural induction of hiPSCs to NPCs ( scale bar = 50 μm).
